# A new Multi Sine-Cosine algorithm for unconstrained optimization problems

**DOI:** 10.1371/journal.pone.0255269

**Published:** 2021-08-06

**Authors:** Muhammad Zubair Rehman, Abdullah Khan, Rozaida Ghazali, Muhammad Aamir, Nazri Mohd Nawi

**Affiliations:** 1 Soft Computing & Data Mining Centre (SMC), Faculty of Computer Science & Information Technology (FSKTM), Universiti Tun Hussein Onn Malaysia, Parit Raja, Malaysia; 2 Institute of Computer Sciences and IT (ICS/IT), The University of Agriculture, Peshawar, Pakistan; Torrens University Australia, AUSTRALIA

## Abstract

The Sine-Cosine algorithm (SCA) is a population-based metaheuristic algorithm utilizing sine and cosine functions to perform search. To enable the search process, SCA incorporates several search parameters. But sometimes, these parameters make the search in SCA vulnerable to local minima/maxima. To overcome this problem, a new Multi Sine-Cosine algorithm (MSCA) is proposed in this paper. MSCA utilizes multiple swarm clusters to diversify & intensify the search in-order to avoid the local minima/maxima problem. Secondly, during update MSCA also checks for better search clusters that offer convergence to global minima effectively. To assess its performance, we tested the MSCA on unimodal, multimodal and composite benchmark functions taken from the literature. Experimental results reveal that the MSCA is statistically superior with regards to convergence as compared to recent state-of-the-art metaheuristic algorithms, including the original SCA.

## Introduction

In modern times, optimization has become pertinent to the development of reliable and robust solutions in the field of science and engineering. Optimization involves certain searching mechanisms that can find the best solutions against an objective function [1]. Mostly, optimization search techniques are categorized into deterministic and stochastic search algorithms. The deterministic algorithms mostly use gradient descent trajectories and extremas to find the most feasible solution. Meanwhile, stochastic search uses multiple points in search space and finds multiple optimal solutions in the search space with more efficacy than the deterministic algorithms [2]. Metaheuristic search mostly employs the stochastic search mechanisms that result in the provision of efficient solutions to non-linear problems [3]. A metaheuristic improvement is grounded in the belief that a stochastic estimate of an optimum will be better than a deterministic solution [2]. Due to their prowess at finding optimal solutions with less computing power, several metaheuristics inspired by nature and physical phenomena have been developed. Some classical examples are differential evolution (DE) [4], genetic algorithm (GA) [5], particle swarm optimization (PSO) [6], ant colony optimization (ACO) [7], cuckoo search (CS) [8], wolf search (WS) [9], artificial bee colony (ABC) [10], bat algorithm (BA) [11], harmony search (HS) [12], and simulated annealing (SA) [13] etc. More recently, a new set of nature-inspired metaheuristics or simply swarm intelligent metaheuristics are developed to solve large-scale optimization problems. These algorithms are grey wolf optimizer (GWO) [14], crow search algorithm (CSA) [15], African buffalo optimization (ABO) [16, 17], whale optimization (WOA) [18], and Sooty-Tern (STOA) [19] etc. The goal of all metaheuristic algorithms is to keep a fine balance between exploration & exploitation of the search space [20]. Although a significant number of algorithms are proposed in this field successfully, but no free lunch (NFL) theorem still welcomes more newbies by suggesting that all algorithms perform optimally in the right environment [3, 21]. Satisfied with NFL theory, Mirjalili et al. proposed a Sine-Cosine algorithm (SCA) in 2015 that uses Sine and Cosine functions for improved metaheuristic search [22]. Despite showing its prowess at solving several optimization problems, it still has problems like low learning curves. Also, its magnitude changes progressively during each iteration that ensues in Sine-Cosine’s early commitment to exploitation which can be counter-productive. Also, in case of many local optimum, Sine-Cosine can converge to a sub-optimal solution [3]. To address the issues faced by the Sine-Cosine algorithm (SCA), this paper proposes an improved Multi Sine Cosine Algorithm (MSCA) that will avoid local optima convergence and improves exploitation. This characteristic makes MSCA suitable for solving optimization problems with multiple local minimums because it maintains a balance between exploration and exploitation with clustered population of solutions. To assess its performance, we adopt the proposed MSCA for the optimization benchmark functions suite in [22]. MSCA’s performance was evaluated against eight popular metaheuristic algorithms, including artificial bee colony (ABC) [23], butterfly optimization algorithm (BOA) [24], crow search algorithm (CSA) [15], differential evolution (DE) [4], grasshopper optimization algorithm (GOA) [25], harmony search (HS) [26], salp swarm optimization (SSA) [27], and the SCA. Experimental results reveal that the MSCA exhibits competitive performance as compared to the SCA and other eight meta-heuristic algorithms. Our contributions are summarized as follows:

- A new Multi sine-cosine algorithm (MSCA) that permits the selection of local and global search operations. In the first stage, MSCA offers clustered population to diversify & intensify the search in order to avoid the local minima. Secondly, during the update, MSCA also checks for better search clusters that offer convergence to global minima effectively. Then, these clusters are merged to form a single cluster *X* that offers a better solution during the search.
- MSCA is tested on 19 complex functions used by Mirjalili in [22]. The optimization problems in these experiments include unimodal, multimodal and composite functions of both low/high dimensions.
- MSCA performed efficiently on hard optimization problems when compared with the state-of-the-art metaheuristic algorithms. Moreover, the efficiency of multiple population division concept of MSCA inspires us to investigate its effect on other metaheuristic algorithms in future studies.

The structure of the paper is organized as follows: Section 2 shed some light on the literature review in which it shed some light on the previous improvements on SCA algorithm; then the original SCA algorithm is discussed in the Sections 3. Section 4 describes the proposed MSCA algorithm. Result and discussions are presented in Section 5. Finally, the paper is concluded in the Section 6.

## Literature review

Owing to the theory of “No free lunch theorem” [28] that makes it impossible for a single algorithm to be a remedy for all optimization problems, Sine-Cosine algorithm (SCA) was proposed in 2016 [22]. SCA works by utilizing the sine and cosine functions to generate a set of interdependent candidate solutions. The new position of the solutions is highly dependent on the previous candidate solution. Since its inception, SCA has been widely utilized on single objective benchmark functions testing [22] and multi-objective functions [29]. Besides benchmark functions, SCA has been applied for solving unit commitment problem in energy production [30] and in feedforward neural networks to predict the liver enzymes of carp fish with high accuracy [31]. Hafez et al. [32] applied SCA for binary feature selection and minimization and to enhance the classification performance. Not satisfied with the convergence in SCA, Elaziz et al. stated that it gets stuck in local minima because the operators used for exploration do not work well [21]. He successfully augmented the performance of SCA with an opposition-based learning platform to generate better solutions. Meshkat et al. came up with an idea of a new weighted update position mechanism (WUPM) instead of the original update method of search agents in SCA. In this method, each search agent was assigned a weight based on its fitness and the position of each agent is updated based on the previous weighted position of the search agent [33]. In 2017, SCA was applied for clustering binarized images of handwritten Arabic text with less noise [34] and for optimization of space shuttle trajectory [35]. A new trend of hybridization was seen at the end of 2017 with the introduction of different high-level heuristics (HLL) and low-level heuristics (LLH) algorithms in SCA. The examples are SCA with Differential Evolution (DE) for structural damage assessment of a truss [36], SCA with Grey Wolf Optimizer (GWO) [37] & SCA with Crow Search for optimization functions [38], Adaptive SCA with Particle swarm optimization (ASCA-PSO) for pairwise local sequence alignment [39], SCA with whale optimization for parameter optimization in a milling process [40], hybrid self-adaptive sine cosine algorithm with opposition based learning [41], and improved sine–cosine algorithm based on orthogonal parallel information for global optimization [42] etc. To find a fine balance between exploration and exploitation, levy flight [43] and chaotic maps [44] were integrated with SCA. Zamli et al. [3] used reinforcement Q-learning table to maintain the states of Sine-Cosine in 2018. They used a reward and punishment mechanism to switch between levy flight and the crossover operator to enhance the solution’s diversity. End of 2018 saw a considerable rise of SCA getting integrated with machine learning techniques such as; parameter enhancement of support vector machines [45], a binary variant of SCA [46], context-based image segmentation [47], breast cancer classification [48], secure data placement in the Internet of Things [49], image thresholding [50], load frequency control of autonomous power system using adaptive fuzzy based PID controller optimized with improved sine cosine algorithm [51] etc.

Later years brought several noteworthy contributions towards the existing SCA algorithm to improve its convergence properties. Most of the proposed improvements were on the modification of exploration and exploitation strategies in SCA. Guo et al., adopted optimal neighborhood and quadratic interpolation strategy to overcome the problem of population update that is guided by the global optimal state in the SCA algorithm. The proposed QISCA used a stochastic optimal neighbor for neighborhood updates, and a quadratic interpolation curve for individual updates. Also, the population’s exploration was enhanced with quasi-opposition learning strategies thus improving the convergence speed and accuracy [52].

In order to improve the exploitation ability of the SCA, a symmetric SCA with adaptive probability selection (SSCA-APS) was introduced. The proposed SSCA searched normally in the early stages using the default parameters. In the later stages, it dynamically adjusted step-sizes of the search with adaptive probability selection (i.e., to integrate original and symmetric sine-cosine operators). Gaussian distribution was used to avoid the local minima by mutating the global optimal individuals in the current generation. A new individual in population is achieved through quasi-interpolation of two randomly selected individuals with a global optimal individual. SSCA-APS was considered a better improvement when tested on benchmark test functions against other SCA variants [53]. Same year, a multi-strategy enhanced SCA algorithm was proposed by Chen et al. to overcome the problem of local optima in SCA for large dimensional problems. The proposed SCA variant employed five strategies (i.e., Cauchy mutation operator, chaotic local search mechanism, opposition-based learning strategy and two operators based on differential evolution) to converge to global optima while maintaining a fine balance between exploration and exploitation. The performance of the proposed SCA variant was verified against other variants on several CEC2014 benchmark and real functions. The simulation results showed that the proposed SCA is better in terms of quality of solutions and convergence speed [54].

Optimal design of off-grid and on-grid hybrid energy management and supply is quite challenging when the energy production is mostly relying on changing climatic conditions. Renewable energy generated from wind turbines are one of the effective carbon emission control strategy of this age. But wind energy’s availability is stochastic in nature. To overcome this problem, Guesmi et al. proposed the integration of chaotic Sine-Cosine algorithm to improve the exploration and exploitation problem of SCA. The improved CSCA algorithm was able to minimize the economic emission dispatch efficiently when applied on the 69-bus ten-unit and 40-unit test systems [55]. Similarly, in another study an improved Sine-cosine with inertial weight algorithm (ISCA) is proposed to design optimal energy management systems for hybrid photovoltaic/wind/fuel cell (PV/WT/FC) system. The target was to minimize the cost of hybrid system life span (CHSLS) for a remote in Iran. Simulation results showed that the ISCA finds easily the optimal combination as PV/WT/FC system with minimal CHSLS than PSO and SCA algorithms [56].

Hydropower systems are another source of clean energy and optimizing operations in hydropower reservoirs can not only help in increased power utilization but also maintain optimal water distribution in arid agricultural zones. Feng et al., tried to answer the reservoir problem with adaptive SCA algorithm. ASCA algorithm used elite mutation strategy to overcome population diversity problem in SCA and the simplex dynamic search strategy to improve solution’s quality in real-time hydropower operations in China [57]. In a similar study by Feng et al., three stage optimization strategy is employed to improve SCA. Quasi-opposition learning strategy is used to find a fine balance between exploration and exploitation. The adaptive mutation is employed in the same manner as in ASCA algorithm. And finally, a random weighting agent generated by multiple leader solutions is integrated into the agent’s evolution equation to improve the overall convergence rate of SCA. The proposed SCA algorithm was tested on several benchmark composite functions. Also, this method showed its mettle in terms of quality of solution and convergence rate when tested on long-term reservoir operations in China [58].

Modern power transmission networks are becoming quite complex due to the integration of several distributed generators (DG). Directional overcurrent relays (DOCR) protect such networks in a highly constrained environment. Sarwagya et al., employed SCA to solve the optimal coordination problems of DOCR on faults generated by 3-bus, 8-bus, 15-bus and 30-bus test systems. SCA was found to effectively reduce the coordination interval time between primary and backup relays [59]. In another similar study, Raut and Mishra proposed multi-objective sine-cosine algorithm for optimal DG allocation. The objectives were to optimally allocate DG in radial distribution systems while minimizing total active power loss, annual energy loss cost, pollutant gas emissions, and maximizing voltage stability index. Their proposed approach was found to be effective; when, it was tested on slightly larger 33-bus and 69-bus distribution systems under four practical load conditions against strength Pareto evolutionary algorithm 2, non-dominated sorting genetic algorithm II, and multi-objective particle swarm optimization [60]. In another study on DG, SCA was successfully used to reduce the effect of global warming and environmental pollution due to the fossil fuel thermal energy generation by integrating wind energy in hydrothermal scheduling (HTS) [61].

With ever growing size of datasets, feature selection offers an efficient way to reduce a dataset’s dimensions and extracting useful information. For this purpose, a multi-objective sine cosine algorithm (SCA) for feature selection (MOSCA_FS) is proposed for hyperspectral imagery. MOSCA_FS is modeled to minimize the redundancy and maximize the relevance of the selected features. MOSCA_FS is found to an effective framework when tested on several benchmark hyperspectral image datasets [62]. To enhance the response of accuracy and response time in machine learning, a new variant called Improved Followers of Salp swarm Algorithm using Sine Cosine algorithm and Disrupt Operator (ISSAFD) is proposed. The ISSAFD works by updating the position of the followers in SSA using sine-cosine algorithm which helps in avoiding local optima altogether [63]. In another high-level hybridization, ABC was effectively integrated with SCA to optimize the threshold values during image segmentation for reduced search region [64]. In the late 2020, SCA was also successfully utilized for reducing features in text categorization in bag of words model [65], and for optimizing local optima problem in Volleyball premier league (VPL) algorithm with high level hybridization [66].

Earlier in 2021, Lawal et al. proposed sine cosine algorithm with artificial neural network (SCA-ANN) models for predicting blast-initiated ground vibrations in five granite mines. The goal was to maximize the safety of human lives and properties near the mining zones by minimizing the impact of vibrations emanating from the blasting of rocks. The proposed SCA-ANN was tested against the Gene expression programming (GEP), adaptive neuro-fuzzy inference system (ANFIS) for predicting the peak particle velocity (PPV). SCA-ANN model trained on 100 datasets and was found to be better than other models with an accuracy of 99 percent with almost zero error [67]. Seeing the intrinsic noise and outlier problems occurring during data clustering in the real world due to initial centroid selection, Kuo et al., proposed a robust SCA-FPCOM algorithm. The proposed SCA-FPCOM originated from the combination of probability c-means, fuzzy c-ordered means, and the sine-Cosine algorithm to improve clustering. The SCA-FPCOM was tested on several datasets and was found to be better than conventional approaches in-terms of adjusted rand index and the Silhouette coefficient [68].

**Fig 1:**
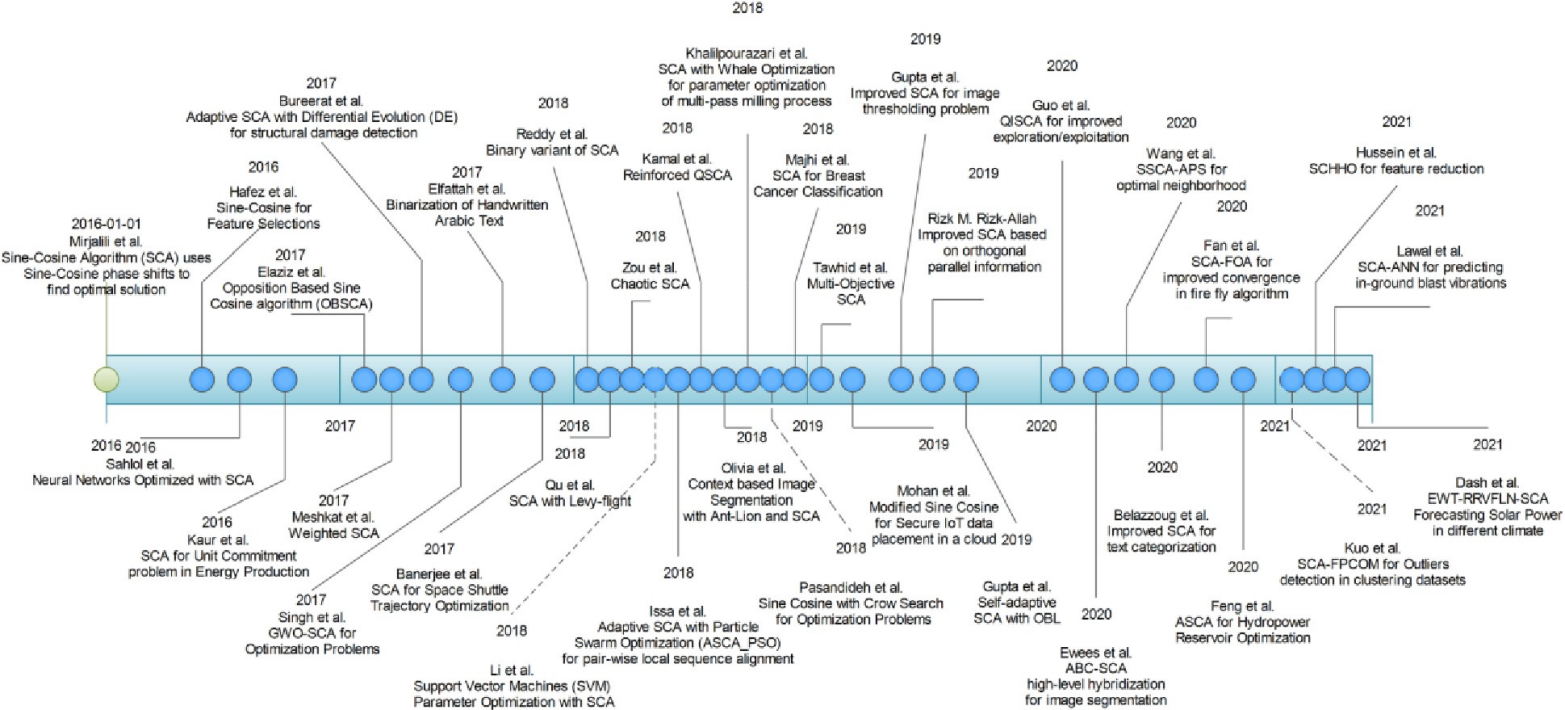
Some of the major contributions on SCA since its inception.

Solar power on-grid generation is becoming a norm these days to avoid generating large electricity bills by selling excess electricity to the grid. Therefore, it is necessary to forecast solar power generation accurately in different weathers. Dash et al., proposed a hybrid forecasting approach consisting of empirical wavelet transform (EWT), Robust minimum variance Random Vector Functional Link Network (RRVFLN) optimized with Sine-Cosine algorithm. The proposed EWT-RRVFLN-SCA was found to be better than the original RRVFLN methods when tested on the historical solar power data [69]. In the mid of 2021, Hussain et al. proposed a hybrid Sine-Cosine Harris Hawk optimization (SCHHO) for minimum feature selection and maximum model generation. Their proposed SCHHO algorithm integrated since-cosine with Harris Hawk optimization to eliminate ineffective exploration in HHO. The SCHHO algorithm was able to adjust exploitation in HHO with dynamic adjustment of candidate solutions thus avoiding stagnancy in local solutions. The SCHHO was tested against state-of-the-art hybrid algorithms on sixteen datasets with high-dimensions exceeding 15000 attributes for numerical optimization. The proposed SCHHO was able to increase convergence speed and reduced features to 87 percent and achieved an accuracy of up to 92 percent [70]. Timeline of some of the most significant contributions to SCA are given in the Fig 1.

## Sine-Cosine algorithm

The Sine-Cosine algorithm (SCA) is a metaheuristic search algorithm proposed by Mirjalili et al. [22]. SCA works by searching solutions in the search based on the sine or cosine function given in Eqs (1) or (2) respectively:

$$X_{i}=X_{i}+r_{1}\times sin(r_{2})\times |r_{3}P_{i}-X_{i}|$$ (1)

$$X_{i}=X_{i}+r_{1}\times cos(r_{2})\times |r_{3}P_{i}-X_{i}|$$ (2)

In general, Both Eqs (1) and (2) are combined into one function as given in the Eq (3):

$$X_{i}=\left\{ \begin{array}{c} X_{i}+r_{1}\times sin(r_{2})\times |r_{3}P_{i}-X_{i}|\;if\;r_{4}<0.5\\ X_{i}+r_{1}\times cos(r_{2})\times |r_{3}P_{i}-X_{i}|\;if\;r_{4}\geq 0.5 \end{array}\right.$$ (3)

**Fig 2:**
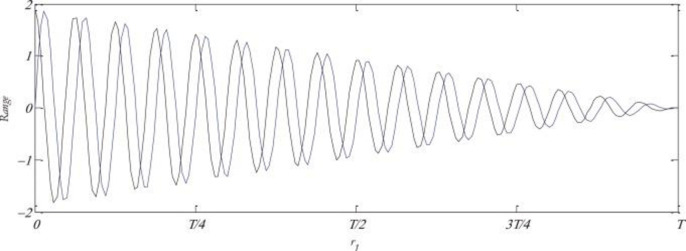
The decreasing pattern of Sine-Cosine indicating a switch from exploration to exploitation in the search space [71].

Where *P*_*i*_ is the destination solution, *X*_*i*_ is the current solution, || indicates the absolute value. *r*_1_, *r*_2_, *r*_3_ and *r*_4_ are the random variables. The parameter *r*_1_ is a random variable responsible for determining the area of the next solution, this area may be either outside space between *X*_*i*_ and *P*_*i*_ or inside them. Mirjalili et al. [22] update the parameter *r*_1_ using the following Equation to balance exploration and exploitation. The effect of *r*_1_ can be seen in the Fig 2.


$$r_{1}=a-t\frac{a}{T}$$ (4)

Where, *a* is a constant, *T* is the maximum number of iterations and *t* is the current iteration.

**Fig 3:**
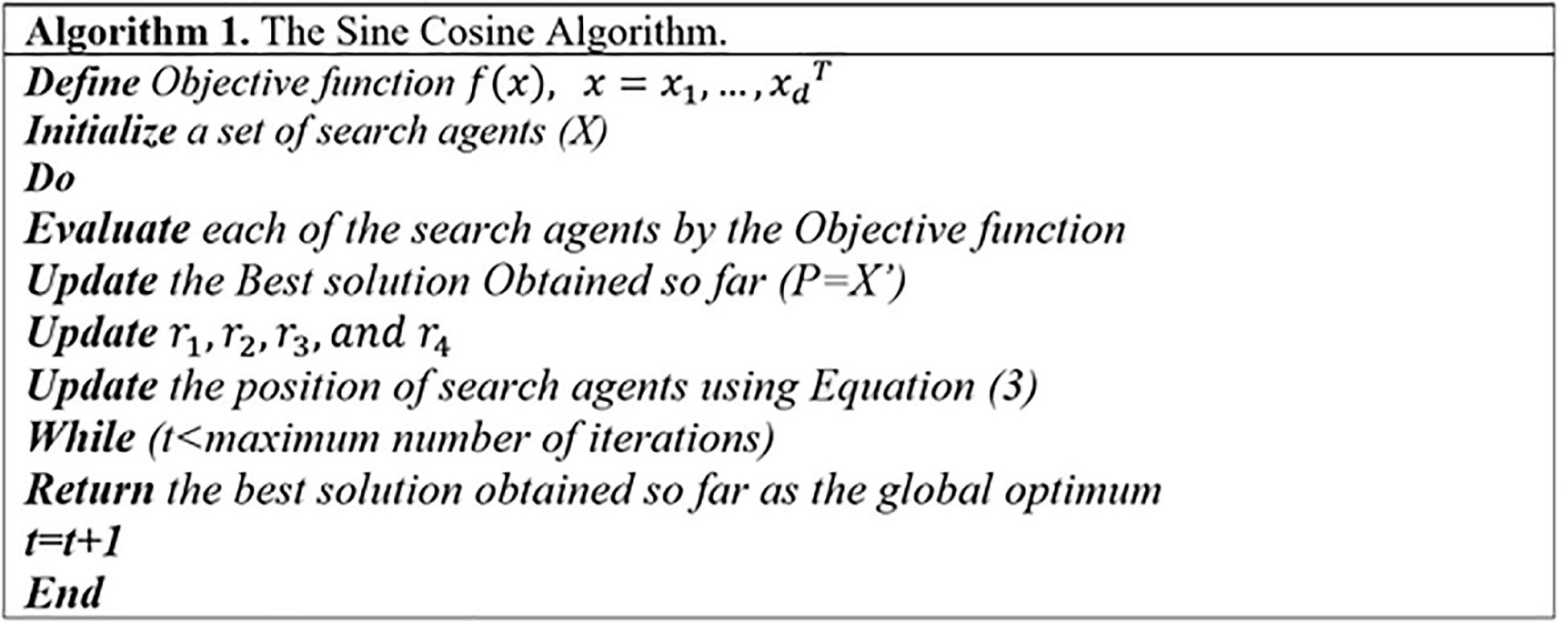
Algorithm 1.

The *r*_2_ is a random variable which used to find the direction of the movement of the next solution (i.e., if it is towards or outwards *P*_*i*_). Also, the *r*_3_ is a random variable which gives random weights for *P*_*i*_ to stochastically emphasize (*r*_3_>1) or de-emphasize (*r*_3_<1) the effect of destination in defining the distance. The *r*_4_ is used to switch between the sine and cosine functions as in Eq (3). The steps of the Sine-Cosine algorithm are given in the Algorithm 1 (Fig 3).

## Improved Multi Sine-Cosine Algorithm (MSCA)

The proposed MSCA algorithm enhances the SCA algorithm in two stages; the first stage offers a clustered population to diversify & intensify the search to avoid the local minima. Secondly, during the update, MSCA also checks for better clusters that offer convergence to global minima effectively. The proposed MSCA algorithm starts by generating random clusters of search agent’s i.e., *X*_1_, *X*_2_, *X*_3_,…,*X*_*n*_ of equal sized population *N*, in which each cluster *X*_*i*_ = [*x*_*i*1_, *x*_*i*2_, *x*_*i*3_,…,*x*_*in*_] represents a complete solution to the specified problem. Then, these clusters are merged together to form a single group *X* that offers a better solution. The steps of the algorithm are as follows;

1. Initialize the random population clusters, *X*_1_, *X*_2_, *X*_3_,…,*X*_*n*_.
2. The value of *r*_1_ in Eq (3) plays a pivot role in MSCA to decide whether to switch from explore or exploit. It gradually decreases from 2 to 0.
3. If, the value of *r*_1_>1, then, MSCA tries to offer better diversity in the new single solution. It merges all the clusters using maximum Euclidean distance (*maxED*) in Eq (5);
$$X_{1,n}=MaxED(X_{1},\ldots ,X_{n})$$ (5)
4. If, the value of *r*_1_<1, then, MSCA tries to offer better intensity in the newly merged single solution by using minimum Euclidean distance (*minED*) in Eq (6);
$$X_{1,n}=MinED(X_{1},{\ldots ,X}_{n})$$ (6)
5. Evaluate each of the search agent clusters by the Objective function (*f(X)*)
6. Update the Best solution out of all clusters Obtained so far (*P = X*)
7. Update *r*_1_, *r*_2_, *r*_3_, *and r*_4_
8. Update the position of search agents using Eqs (5 and 6)
9. Continue evaluating against the Objective function until all the conditions are met or the iterations are finished

**Fig 4:**
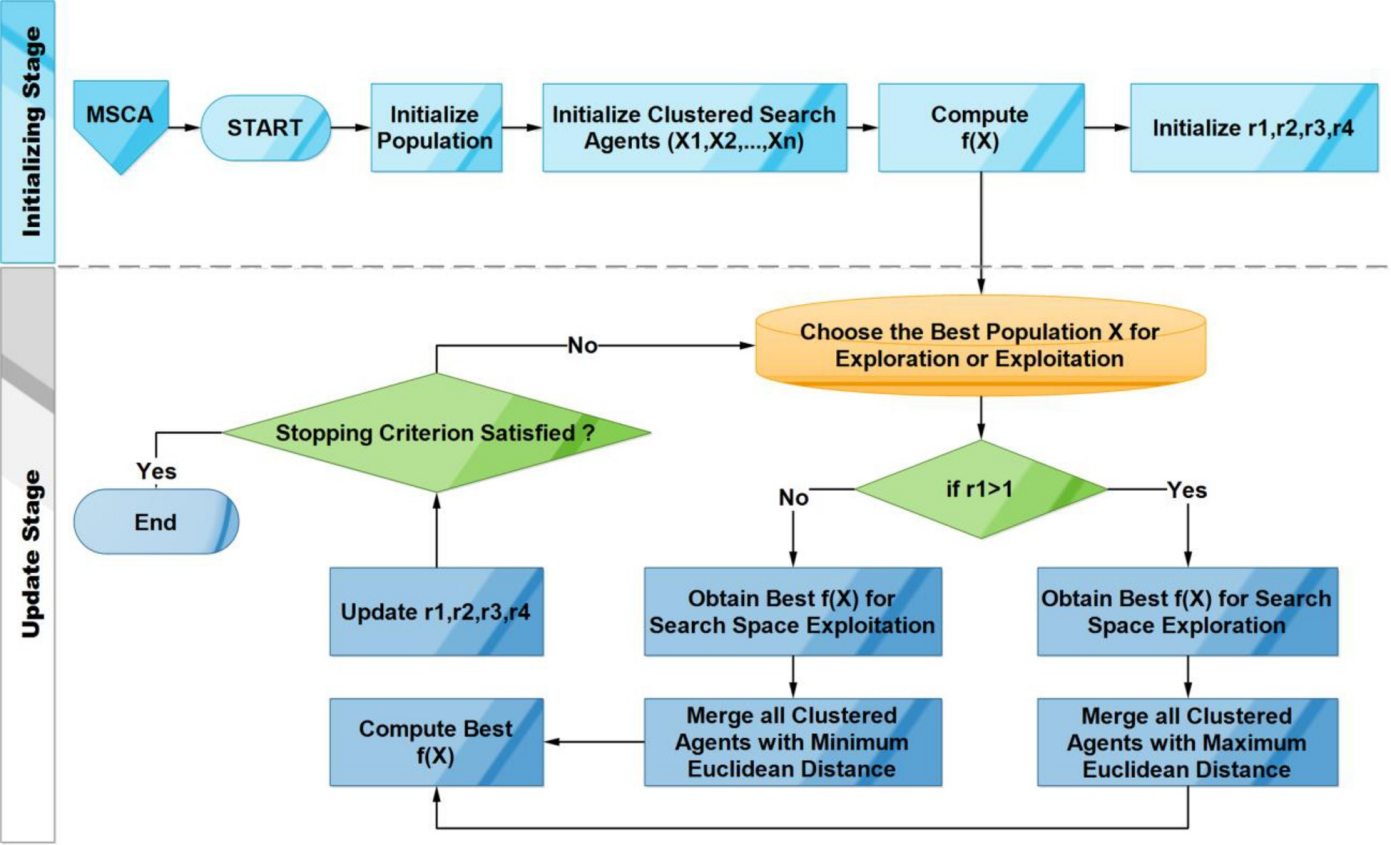
The workflow of the proposed MSCA algorithm.

The proposed Multi Sine-Cosine algorithm is demonstrated in Fig 4.

## Results and discussions

In this section, MSCA is tested on benchmark functions and its performance is compared with eight algorithms. The detailed implementation, parameter settings, and results are discussed in the sub-sections.

### Implementation of MSCA and the benchmark algorithms

The proposed MSCA algorithm’s performance is evaluated on the benchmark functions commonly used by Mirjalili et al. [71]. The system used for simulations was an Intel Core i5 processor with 8GB of RAM. The proposed MSCA was implemented on MATLAB R2018b with Windows 10 and compared with the latest and most popular algorithms given in Table 1. Three types of benchmark functions were used to evaluate the efficiency of the proposed MSCA algorithm, i.e., unimodal, multimodal, and composite. The detailed descriptions of the function and their properties are given in the sub-sections.

**Table 1:** Initialization parameter values for the comparison algorithms.

Algorithm (s)	Parameter (s)
**Artificial Bee Colony (ABC)**	Bee Colony Size = 30
	Acceleration Coefficient Upper Bound = 1
	Number of Decision Variables = 2
**Butterfly Optimization Algorithm (BOA)**	Number of Butterflies = 30
	Probability switch = 0.8
	Power Exponent = 0.1
	Sensory Modality = 0.01
**Crow Search Algorithm (CSA)**	Number of Crows = 30
	Awareness Probability = 0.2
**Differential Evolution (DE)**	Population Size = 30
	Lower Bound of Scaling Factor = 0.2
	Upper Bound of Scaling Vector = 0.8
	Crossover probability = 0.2
**Grasshopper Optimization Algorithm (GOA)**	No of Grass-Hoppers = 30
	cMax = 1
	cMin = 0.00004
**Harmony Search (HS)**	Harmony Memory Size = 30
	Number of New Harmonies = 30
	Harmony Memory Consideration Rate = 0.9
	Pitch Adjustment Rate = 0.1
**Salp Swarm Algorithm (SSA)**	Search agents = 30
	c1 = linearly decreases from 2 to 0
**Sine-Cosine Algorithm (SCA)**	Search Agents = 30
	a = linearly decreases from 2 to 0
**Multi Sine-Cosine Algorithm (MSCA)**	Search Agents = 30
	a = linearly decreases from 2 to 0
	Cluster Numbers = 6 with each cluster containing equally divided search agents

### Parameter settings

During all the experiments, default parameters were used for all the parallel algorithms. Meanwhile, the proposed MSCA algorithm used a population size of 30 that was equally divided among all six clusters. For a fair comparison, the maximum iterations for all the algorithms were set to 1000 with 30 trials on each function. The parameter setting of all the algorithms used in this paper is given in Table 1.

### Statistical analysis

For statistical analysis, Standard deviation (SD) is used to find any variations in the average trial values and Mean formula is used to calculate the average of all trials. The equations of SD and Mean are expressed as [2];

$$SD=\sqrt{\frac{\sum_{i=1}^{n}{\left(x_{i}-x\prime \right)}^{2}}{n-1}}$$ (7)

$$Mean=\frac{x_{1}+\cdots +x_{n}}{N}$$ (8)

Where *n* is the total number of inputs, *N* is the total number of values or elements, *x*_*i*_ is the number of input patterns, and *x*′ is the mean of *x*_*i*_.

Sometimes, it is not fair to say that the proposed algorithm is better because it performed statistically better in terms of mean and SD over 30 independent trials as SD and mean does not compare each run. Therefore, to decide on the significance of each result, the Wilcoxon rank-sum is used to find whether the proposed algorithm is statistically better in performance than the rest of the algorithms [72]. The null hypothesis *H*_0_ shows that there is no significant difference as far as the sample size is concerned for MSCA and each comparison algorithm. Alternative hypothesis, *H*_1_ means that MSCA’s sample size is less than that of each comparison algorithm [13].

### Description of the benchmark functions

**Fig 5:**
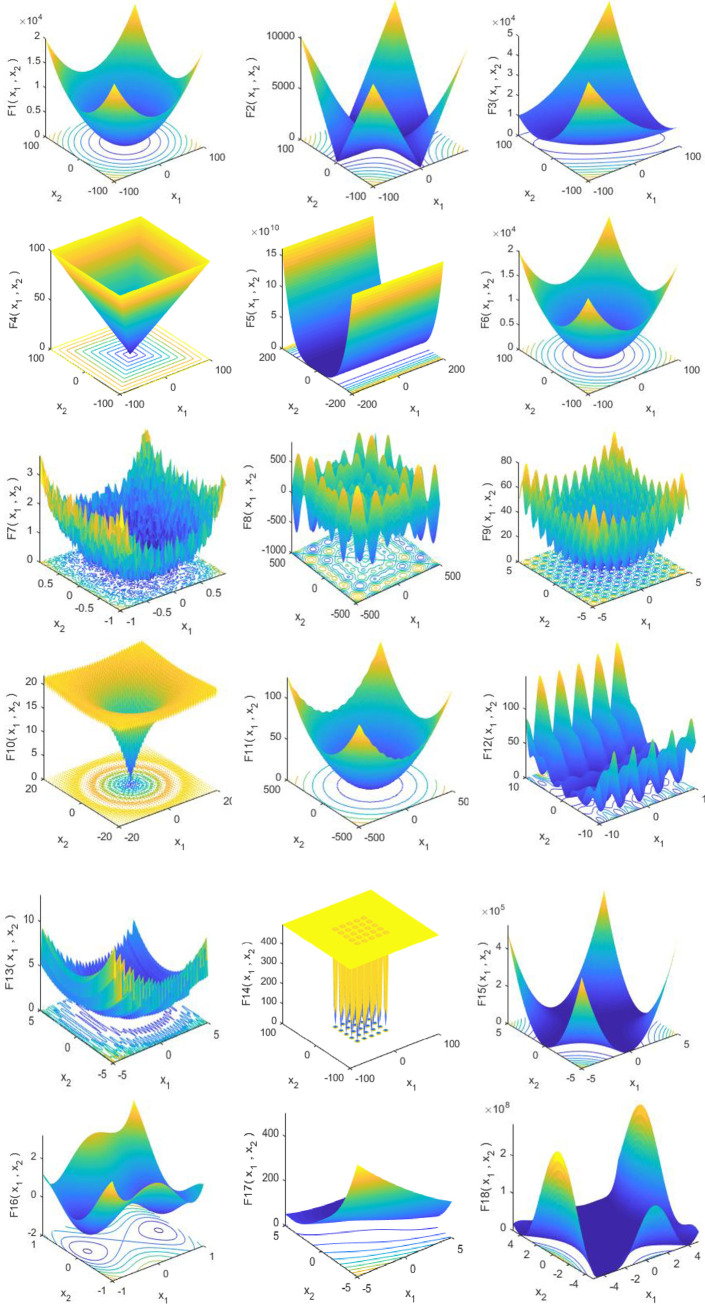
Two-dimensional (2-D) view of the benchmark functions.

The proposed MSCA algorithm is evaluated based on the 19 mathematical functions used by Mirjalili et al. [71]. For testing the algorithms, the benchmark functions are divided into three complex types; i.e., Unimodal, multimodal, and composite functions etc. The two-dimensional (2-D) view of the functions is illustrated in the Fig 5.

The detailed descriptions of the function and their properties are given in Tables 2 and 3. Dimension, Range, and *f*_*min*_ columns in the Tables 2 and 3 denote the dimensions of the function, boundary of the search space, and the cost of the function.

**Table 2:** Mathematical formulae of the benchmark functions.

Function (s)	Mathematical Formula
**F01**	$f(x)=\sum_{i=1}^{n}x_{i}^{2}$
**F02**	$f(x)=\sum_{i=1}^{n}|x_{i}|+\prod_{i=1}^{n}\left|x_{i}\right|$
**F03**	$f(x)=\sum_{i=1}^{n}{\left(\sum_{j=1}^{i}x_{j}\right)}^{2}$
**F04**	*f*(*x*) = *max*{|*x*_*i*_|, 1≤*i*≤*n*}
**F05**	$f(x)=\sum_{i=1}^{n}\left[100{\left(x_{i+1}-x_{i}^{2}\right)}^{2}+{\left(x_{i}-1\right)}^{2}\right]$
**F06**	$f(x)=\sum_{i=1}^{n}{\left({|x}_{i}+0.5|\right)}^{2}$
**F07**	$f(x)=\sum_{i=1}^{n}ix_{i}^{4}+random(0,1)$
**F08**	$f(x)=\sum_{i=1}^{n}-x_{i}sin(\sqrt{\left|x_{i}\right|})$
**F09**	$f(x)=\sum_{i=1}^{n}\left[x_{i}^{2}-10cos(2\pi x_{i})+10\right]$
**F10**	$f(x)=-20exp\left(-0.2\sqrt{\frac{1}{n}\sum_{i=1}^{n}x_{i}^{2}}\right)-exp\left(\frac{1}{n}\sum_{i=1}^{n}cos(2\pi x_{i}\right)+20+e$
**F11**	$f(x)=\frac{1}{4000}\sum_{i=1}^{n}x_{i}^{2}-\prod_{i=1}^{n}cos(\frac{x_{i}}{\sqrt{1}})+1$
**F12**	$f(x)=\frac{\pi }{n}\left\{10sin(\pi y_{1})+\sum_{i=1}^{n-1}{\left(y_{i}-1\right)}^{2}[1+10{sin}^{2}({\pi y}_{i+1})]+{\left(y_{n}-1\right)}^{2}\right\}+\sum_{i=1}^{n}u(x_{i},10,100,4)$ $y_{i}=1+\frac{x_{i}+1}{4}$ $u(x_{i},a,k,m)=\{\begin{array}{c} k{\left(x_{i}-a\right)}^{m}x_{i}>a\\ 0-a<x_{i}<a\\ k{\left(-x_{i}-a\right)}^{m}<x_{i}<a \end{array}$
**F13**	$f(x)=0.1\left\{{sin}^{2}(3\pi x_{1})+\sum_{i=1}^{n}\left({x_{i}-1)}^{2}[1+{sin}^{2}(3\pi x_{i}+1)]+{\left(x_{n}-1\right)}^{2}[1+{sin}^{2}(2\pi x_{n})\right]\right\}+\sum_{i=1}^{n}u(x_{i},5,100,4)$
**F14**	*f*(*CF*1): *f*_1_, *f*_2_, *f*_3_,…,*f*_10_ = *Sphere Function* [*σ*_1_, *σ*_2_, *σ*_3_,…,*σ*_10_] = [1,1,1,…,1] $\left[\lambda_{1},\lambda_{2},\lambda_{3},\ldots ,\lambda_{10}\right]=\left[\frac{5}{100},\frac{5}{100},\frac{5}{100},\ldots ,\frac{5}{100}\right]$
**F15**	*f*(*CF*2): *f*_1_, *f*_2_, *f*_3_,…,*f*_10_ = *Griewank′s Function* [*σ*_1_, *σ*_2_, *σ*_3_,…,*σ*_10_] = [1,1,1,…,1] $[\lambda_{1},\lambda_{2},\lambda_{3},\ldots ,\lambda_{10}]=\left[\frac{5}{100},\frac{5}{100},\frac{5}{100},\ldots ,\frac{5}{100}\right]$
**F16**	*f*(*CF*3): *f*_1_, *f*_2_, *f*_3_,…,*f*_10_ = *Griewank′s Function* [*σ*_1_, *σ*_2_, *σ*_3_,…,*σ*_10_] = [1,1,1,…,1] [*λ*_1_, *λ*_2_, *λ*_3_,…,*λ*_10_] = [1,1,1,…,1]
**F17**	*f*(*CF*4): *f*_1_, *f*_2_ = *Ackley′s Function* *f*_3_, *f*_4_ = *Rastrigin′s Function* *f*_5_, *f*_6_ = *Weirstrass Function* *f*_7_, *f*_8_ = *Griewank′s Function* *f*_9_, *f*_10_ = *Sphere Function* [*σ*_1_, *σ*_2_, *σ*_3_,…,*σ*_10_] = [1,1,1,…,1] $[\lambda_{1},\lambda_{2},\lambda_{3},\ldots ,\lambda_{10}]=\left[\frac{5}{32},\frac{5}{32},1,1,\frac{5}{0.5},\frac{5}{0.5},\frac{5}{100},\frac{5}{100},\frac{5}{100},\frac{5}{100}\right]$
**F18**	*f*(*CF*5): *f*_1_, *f*_2_ = *Rastrigin′s Function* *f*_3_, *f*_4_ = *Weirstrass Function* *f*_5_, *f*_6_ = *Griewank′s Function* *f*_7_, *f*_8_ = *Ackley′s Function* *f*_9_, *f*_10_ = *Sphere Function* [*σ*_1_, *σ*_2_, *σ*_3_,…,*σ*_10_] = [1,1,1,…,1] $[\lambda_{1},\lambda_{2},\lambda_{3},\ldots ,\lambda_{10}]=\left[\frac{1}{5},\frac{1}{5},\frac{5}{0.5},\frac{5}{0.5},\frac{5}{100},\frac{5}{100},\frac{5}{32},\frac{5}{32},\frac{5}{100},\frac{5}{100}\right]$
**F19**	*f*(*CF*6): *f*_1_, *f*_2_ = *Rastrigin′s Function* *f*_3_, *f*_4_ = *Weirstrass Function* *f*_5_, *f*_6_ = *Griewank′s Function* *f*_7_, *f*_8_ = *Ackley′s Function* *f*_9_, *f*_10_ = *Sphere Function* [*σ*_1_, *σ*_2_, *σ*_3_,…,*σ*_10_] = [0.1,0.2,0.3,0.4,0.5,0.6,0.7,0.8,0.9,1] [*λ*_1_, *λ*_2_, *λ*_3_,…,*λ*_10_] $=\left[0.1*\frac{1}{5},0.2*\frac{1}{5},0.3*\frac{5}{0.5},0.4*\frac{5}{0.5},0.5*\frac{5}{100},0.6*\frac{5}{100},0.7*\frac{5}{32},0.8*\frac{5}{32},0.9*\frac{5}{100},1*\frac{5}{100}\right]$

**Table 3:** Properties of the benchmark functions.

Function (s)	Dimension	Range	*f*_*min*_
**F01**	20	[−100,100]	0
**F02**	20	[−10,10]	0
**F03**	20	[−100,100]	0
**F04**	20	[−100,100]	0
**F05**	20	[−30,30]	0
**F06**	20	[−100,100]	0
**F07**	20	[−128,1.28]	0
**F08**	20	[−500,500]	-2094.98
**F09**	20	[−5.12,5.12]	0
**F10**	20	[−32,32]	0
**F11**	20	[−600,600]	0
**F12**	20	[−50,50]	0
**F13**	20	[−50,50]	0
**F14**	10	[−5,5]	0
**F15**	10	[−5,5]	0
**F16**	10	[−5,5]	0
**F17**	10	[−5,5]	0
**F18**	10	[−5,5]	0
**F19**	10	[−5,5]	0

### Parametric test analysis

Depending on the complexity of the benchmark functions, they are divided into three types; i.e., Unimodal (F01-F06), multimodal (F07-F13), and composite (F14-F19). Unimodal functions have no local optima, a single global optimum and used to evaluate the exploitation capability of the algorithm. Multimodal functions have multiple local optima, a single global optimum and used to measure the exploration capability of the algorithm. Meanwhile, the composite functions are used to find the fine balance between exploration and exploitation capability of an algorithm. The statistical mean, SD and CPU time are given in the Tables 4–6 respectively.

**Table 4:** Average mean values by algorithms on benchmark functions.

Functions	ABC	BOA	CSA	DE	GOA	HS	SSA	SCA	MSCA
**F01**	6.42E-28	3.49E-12	4.87E-08	1.09E-22	5.19E-03	7.28E-09	1.35E-08	1.29E-27	1.81E-36
**F02**	2.20E-05	1.07E-11	1.27E+00	4.84E-13	1.56E-07	8.87E-05	1.96E-05	5.28E-20	2.97E-20
**F03**	3.16E-07	1.67E-14	1.14E-05	2.88E-07	3.78E-08	1.78E-10	1.81E-09	1.16E-11	5.24E-21
**F04**	2.39E-02	1.13E-11	5.66E-03	2.48E-03	1.75E-05	2.27E-03	1.82E-05	1.14E-07	2.55E-11
**F05**	3.50E+00	8.92E+00	1.69E+07	1.47E+01	4.84E+00	7.02E-01	6.98E+00	8.25E+00	7.97E+00
**F06**	2.01E-07	1.26E+00	4.22E-07	9.78E-23	2.42E-10	7.61E-09	7.35E-10	3.91E-01	1.68E-01
**F07**	2.28E-03	3.67E-03	2.02E-02	1.45E-02	2.60E-01	4.89E-03	4.11E-03	2.16E-03	6.73E-04
**F08**	-2948.75	-1910.08	-478.00	-78.71	-1439.22	-19.73	-3243.35	-2052.04	-2084.98
**F09**	3.48E+00	3.65E+01	4.5E+01	9.40E-02	2.6E+01	3.96E-06	1.6E+01	3.8E-05	1.04E-11
**F10**	1.31E-03	6.66E-13	2.30E+00	6.12E-12	1.54E-06	2.76E-04	5.60E-01	3.50E-06	7.06E-15
**F11**	7.76E-02	4.11E-01	1.12E-01	0.00E+00	1.34E-01	1.38E-02	2.48E-01	1.15E-01	1.90E-11
**F12**	4.47E-08	5.76E-02	3.79E+00	1.89E-24	4.88E-09	8.58E-09	2.52E-01	2.03E-01	2.42E-03
**F13**	5.16E-03	3.94E-02	2.25E+01	4.08E-02	2.85E-02	2.46E-02	3.76E-02	3.04E-02	3.98E-03
**F14**	7.87E+00	9.98E-01	9.98E-01	2.98E+00	1.56E+01	9.98E-01	9.98E-01	9.98E-01	9.98E-01
**F15**	2.06E-03	5.10E-04	1.82E-02	7.07E-04	3.96E-02	3.89E-03	4.77E-03	1.80E-03	3.83E-04
**F16**	-1.03E+00	-0.01E+02	1.26E+00	-1.03E+00	-1.03E+00	-1.03E+00	-1.03E+00	-1.03E+00	-1.03E+00
**F17**	3.98E-01	3.98E-01	4.81E+00	3.98E-01	3.98E-01	3.98E-01	-3.20E+00	3.98E-01	3.98E-01
**F18**	3.00E+00	3.53E+01	8.02E+03	3.00E+00	3.00E+00	3.00E+00	3.00E+00	3.00E+00	3.00E+00
**F19**	-3.86E+00	-3.86E+00	-1.10E+00	-3.86E+00	-2.80E+00	-3.86E+00	-3.86E+00	-3.84E+00	-3.71E+00

**Table 5:** Standard deviation (SD) of algorithms on benchmark functions.

Functions	ABC	BOA	CSA	DE	GOA	HS	SSA	SCA	MSCA
**F01**	9.88E-29	6.17E-13	4.13E-08	8.04E-23	3.92E-03	8.5E-09	2.52E-09	2.26E-27	3.59E-36
**F02**	1.04E-05	6.53E-13	1.07E+00	1.03E-13	2.44E-07	2.9E-05	3.29E-05	5.24E-20	2.67E-20
**F03**	1.67E-07	1.44E-15	1.98E-05	3.69E-07	7.26E-08	8.2E-11	6.29E-10	1.40E-11	2.89E-21
**F04**	2.39E-02	8.21E-13	3.79E-03	3.22E-04	4.18E-06	3.1E-03	4.11E-06	2.54E-07	1.97E-11
**F05**	3.16E+00	7.05E-03	3.02E+07	1.04E+00	1.00E+01	5.1E-01	1.78E+00	2.05E+00	3.26E-01
**F06**	1.92E-07	2.49E-01	2.43E-07	5.74E-23	1.71E-10	4.8E-09	1.90E-10	3.94E-02	1.24E-01
**F07**	1.13E-03	3.93E-03	1.63E-02	2.21E-03	3.45E-01	5.4E-03	1.86E-03	1.36E-03	6.86E-04
**F08**	2.19E+03	2.56E+02	7.42E+01	0.00E+00	1.95E+02	0.0E+00	1.83E+02	70.87	1.51E+02
**F09**	1.15E+00	5.99E+00	1.95E+01	1.63E-01	3.69E+00	3.6E-06	7.66E+00	8.53E-05	1.04E-11
**F10**	5.65E-04	9.96E-13	9.29E-01	1.00E-12	7.07E-07	1.4E-04	7.87E-01	4.47E-06	7.59E-15
**F11**	3.04E-03	3.12E-01	7.37E-02	0.00E+00	6.41E-02	4.5E-03	1.30E-01	1.51E-01	2.08E-11
**F12**	1.28E-08	3.07E-02	3.08E+00	9.38E-25	5.49E-09	9.6E-09	7.45E-02	8.12E-02	1.05E-03
**F13**	3.05E-03	3.40E-02	3.63E+01	3.83E-02	2.38E-02	1.2E-02	2.84E-02	4.42E-02	3.00E-03
**F14**	0.00E+00	0.00E+00	0.00E+00	0.00E+00	6.83E+00	0.0E+00	0.00E+00	0.00E+00	0.00E+00
**F15**	2.44E-03	2.62E-04	2.26E-02	7.83E-05	4.14E-02	4.6E-03	3.30E-03	7.92E-04	3.31E-04
**F16**	0.00E+00	1.55E+03	8.72E-01	0.00E+00	0.00E+00	0.0E+00	0.00E+00	0.00E+00	0.00E+00
**F17**	0.00E+00	0.00E+00	1.52E+00	0.00E+00	0.00E+00	0.0E+00	0.00E+00	6.67E-04	0.00E+00
**F18**	0.00E+00	0.00E+00	9.28E+03	0.00E+00	0.00E+00	0.0E+00	0.00E+00	0.00E+00	0.00E+00
**F19**	3.25E-03	9.86E-04	5.45E-01	4.97E-16	7.81E-01	5.0E-16	0.00E+00	7.16E-03	2.19E-01

**Table 6:** Elapsed time of algorithms on benchmark functions.

Functions	ABC	BOA	CSA	DE	GOA	HS	SSA	SCA	MSCA
**F01**	6.37	2.61	3.79	8.95	14.40	5.05	4.16	4.12	4.84
**F02**	7.17	3.11	3.83	6.73	13.73	5.65	4.22	4.36	4.91
**F03**	7.79	2.91	3.87	7.45	15.37	5.04	4.15	4.53	4.37
**F04**	7.39	4.59	3.88	5.59	14.70	4.04	4.19	4.19	4.63
**F05**	8.58	5.25	3.67	5.74	13.79	4.12	4.11	4.36	5.05
**F06**	7.04	2.99	3.44	5.61	14.67	3.94	4.86	4.44	5.09
**F07**	8.41	4.46	4.01	6.10	13.18	4.20	4.20	4.53	4.14
**F08**	7.93	5.79	5.03	5.54	15.06	4.32	4.22	3.44	4.09
**F09**	6.88	3.24	3.93	5.86	14.22	3.92	4.22	4.20	4.33
**F10**	7.10	3.58	3.31	7.05	13.74	4.11	4.75	4.18	4.21
**F11**	7.38	2.48	3.71	5.65	15.61	4.49	4.32	2.90	4.61
**F12**	11.02	5.23	4.32	7.27	16.99	4.61	4.24	3.90	4.93
**F13**	3.48	5.40	3.96	5.76	2.86	6.39	4.56	5.12	4.54
**F14**	5.13	10.15	3.35	6.64	3.24	6.64	4.28	5.44	4.16
**F15**	4.26	4.56	3.87	6.38	2.15	6.51	4.30	5.66	4.18
**F16**	10.29	2.88	3.78	5.90	2.17	5.70	4.32	4.97	4.20
**F17**	9.88	5.47	3.98	6.08	2.14	5.69	4.39	5.32	4.20
**F18**	9.03	2.43	3.01	6.19	2.49	6.16	4.44	5.02	4.27
**F19**	15.03	2.97	2.78	6.82	2.15	5.14	4.50	5.67	4.19

**Fig 6:**
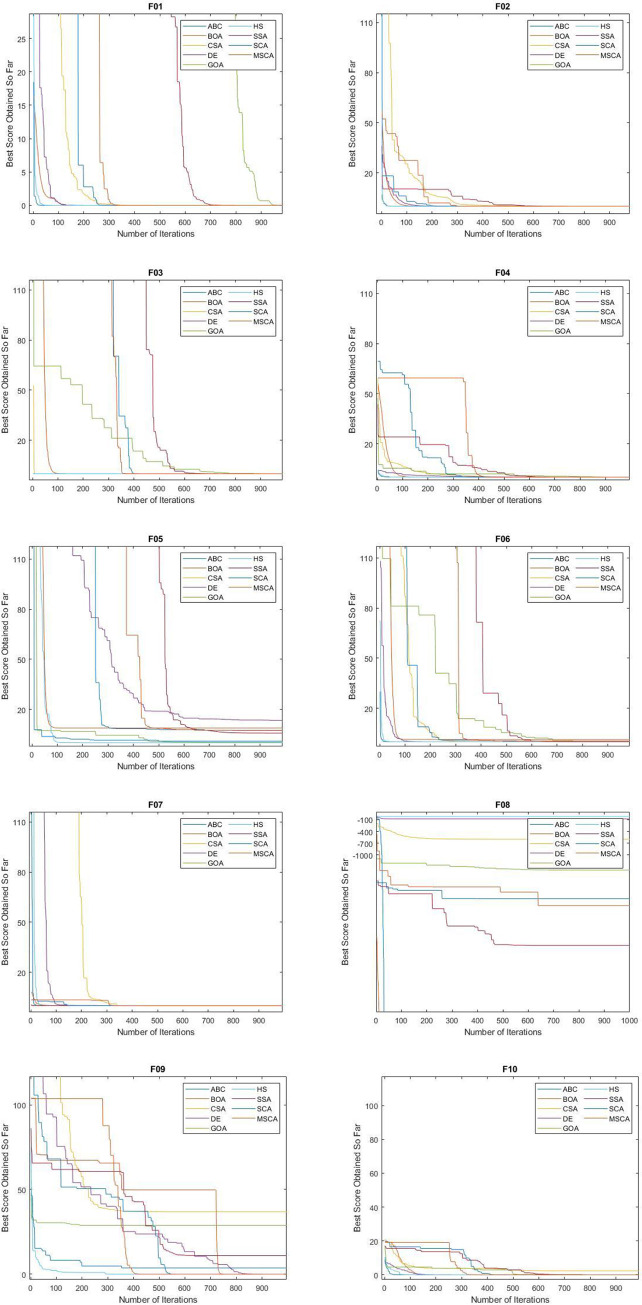
Convergence performance of MSCA algorithm on unimodal and multimodal functions.

**Fig 7:**
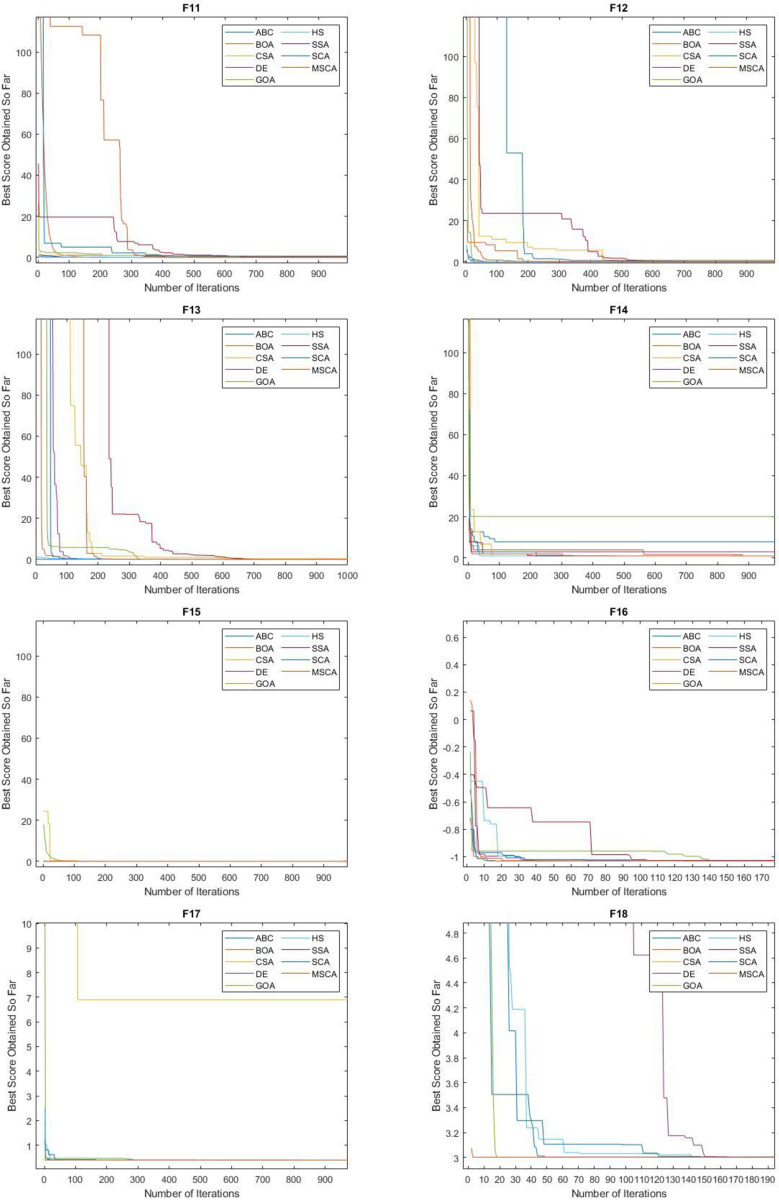
Convergence performance of MSCA algorithm on multimodal and composite functions.

From Table 5, we can witness that the MSCA gained the best mean values on F1-F3, F7-F11, F13-F15, and F17-F19. Meanwhile, the second-best mean values were observed for ABC on F01- F04, F11, F13, F14, and F16-F18. Third best mean values were recorded on SCA for F01-F04, F07-F11, and BOA for F03-F04, F06, F08, F10, F15, F18. Best values were shown by BOA for F04, HS for F05, and DE for F06. Overall, it can be seen that MSCA has high searching precision on unimodal and multimodal problems. Despite the complexity they offer during search by composite functions, MSCA performed better. The CSA was found to be the most unstable algorithm in the above batch and was not able to perform on multimodal or composite functions. The mean convergence performance of all the algorithms can be seen in the Figs 6 and 7.

For standard deviation (SD), MSCA is able to perform with best SD values on F01-F03, F07, F09-F10, F13-F14, and F16-F18. Similarly, BOA showed best results on F04-F05, DE on F06, F11-F12, F14-F16, and HS on F08, F14, F16. Although, most of the algorithms showed stable SD on majority of the functions but ABC was the most stable among them. Again, worst deviation in results were shown by CSA. All SD values can be seen in the Table 6.

In recent age, CPU time is becoming negligible because of the ever-increasing speed of CPU’s but still it was recorded for all algorithm and results are illustrated in the Table 6. Here, BOA took the best average time on most functions i.e., F01-F03, and F09. CSA took the crown of best average time on most of the functions i.e., F04-F07, and F10-F11, followed by GOA which only performed on F13-F19, SCA on F12, and the proposed MSCA on F08.

### Non-parametric test analysis

In this paper, the Wilcoxon rank-sum test is used to determine the significance of the results obtained by MSCA with five other algorithms. Table 7 shows the p-values obtained by MSCA and SCA. The superior significance of the proposed MSCA can be seen in Table 7, where it outperforms SCA, SSA, HS, GOA, and DE algorithms.

**Table 7:** Wilcoxon rank-sum test results over all runs.

Functions	MSCA Vs. SCA	MSCA Vs. SSA	MSCA Vs. HS	MSCA Vs. GOA	MSCA Vs. DE
	P value	P value	P value	P value	P value
**F01**	0.0022	2.31E-04	1.33E-07	1.12E-06	0.0022
**F02**	0.5887	1.39E-04	4.22E-07	2.87E-07	0.0022
**F03**	0.0022	0.0022	1.33E-07	1.33E-06	3.97E-04
**F04**	0.0022	0.0022	1.11E-07	3.97E-04	1.39E-05
**F05**	0.0022	0.512	0.133	0.133	0.011
**F06**	0.0260	0.339	0.111	0.23	1.343
**F07**	0.0260	3.97E-03	0.0197	0.0022	0.0022
**F08**	0.0022	0.0022	0.0197	0.01	0.0022
**F09**	0.0022	0.0022	0.0022	1.39E-05	3.97E-04
**F10**	0.0022	0.0022	0.0022	3.97E-04	0.0022
**F11**	0.0022	0.0022	0.0022	3.97E-04	1.39E-05
**F12**	0.0022	0.0022	0.511	0.113	0.212
**F13**	0.0411	0.0197	0.0022	0.0022	0.0197
**F14**	N/A	N/A	N/A	N/A	N/A
**F15**	0.0022	0.0022	0.0022	0.0197	0.512
**F16**	N/A	N/A	N/A	N/A	N/A
**F17**	N/A	N/A	N/A	N/A	N/A
**F18**	N/A	N/A	N/A	N/A	N/A
**F19**	0.0022	0.0022	0.0022	0.0022	0.0022

## Conclusions

A new Multi Sine Cosine algorithm (MSCA) is introduced in this paper. The original Sine Cosine algorithm had a problem of early commitment to exploitation that leads it towards sub-optimal solutions. The proposed MSCA adopts the method of population clusters to diversify & intensify the search in order to avoid the local minima. Secondly, during the update, MSCA also checks for better search clusters that offer convergence to global minima effectively. The proposed MSCA avoids the premature convergence to local optima and avoids variance during convergence. MSCA’s performance is evaluated against eight popular metaheuristic algorithms, including ABC, BOA, CSA, DE, GOA, HS, SSA, and the SCA on 19 complex benchmark functions. Experimental results reveal that the MSCA exhibits competitive performance as compared to the SCA and other eight meta-heuristic algorithms. MSCA performed efficiently on hard optimization problems when compared with the state-of-the-art metaheuristic algorithms. Moreover, the efficiency of multiple population division concept of MSCA inspires us to investigate its effect on other metaheuristic algorithms in the future studies.

## Supporting information

S1 Dataset Benchmark functions/ datasets used in this paper. (DOCX)
