# Supplementary material for: A new Multi Sine-Cosine algorithm for unconstrained optimization problems
Source: PLoS One. 2021 Aug 6;16(8):e0255269. doi: 10.1371/journal.pone.0255269 (PMC8345889; doi:10.1371/journal.pone.0255269)
Supplement: S1 Dataset — (DOCX) [file pone.0255269.s001.docx]

S1 Dataset. Benchmark Functions/ Datasets used in this paper

Table 2. Mathematical Formulae of the Benchmark Functions

| **Function (s)** | **Mathematical Formula** |
| --- | --- |
| **F01** | $f\left( x \right)=\sum_{i=1}^{n} x_{i}^{2}$ |
| **F02** | $f\left( x \right)=\sum_{i=1}^{n} \left\vert x_{i} \right\vert+\prod_{i=1}^{n} \vert x_{i}\vert$ |
| **F03** | $f\left( x \right)=\sum_{i=1}^{n} \left( \sum_{j=1}^{i} x_{j} \right)^{2}$ |
| **F04** | $f\left( x \right)=max\{\left\vert x_{i} \right\vert, 1\leq i\leq n\}$ |
| **F05** | $f\left( x \right)=\sum_{i=1}^{n} [100\left( x_{i+1}-x_{i}^{2} \right)^{2}+\left( x_{i}-1 \right)^{2}]$ |
| **F06** | $f\left( x \right)=\sum_{i=1}^{n} \left( {\vert x}_{i}+0.5\vert\right)^{2}$ |
| **F07** | $f\left( x \right)=\sum_{i=1}^{n} ix_{i}^{4}+random(0,1)$ |
| **F08** | $f\left( x \right)=\sum_{i=1}^{n} -x_{i}sin(\sqrt{\vert x_{i}\vert})$ |
| **F09** | $f\left( x \right)=\sum_{i=1}^{n} [x_{i}^{2}-10cos\left( 2\pi x_{i} \right)+10]$ |
| **F10** | $f\left( x \right)=-20exp\left( -0.2\sqrt{\frac{1}{n}\sum_{i=1}^{n} x_{i}^{2}} \right)-exp\left( \frac{1}{n}\sum_{i=1}^{n} cos(2\pi x_{i} \right)+20+e$ |
| **F11** | $f\left( x \right)=\frac{1}{4000}\sum_{i=1}^{n} x_{i}^{2}-\prod_{i=1}^{n} cos\left( \frac{x_{i}}{\sqrt{1}} \right)+1$ |
| **F12** | $f\left( x \right)=\frac{\pi}{n}\left\{ 10sin\left( \pi y_{1} \right)+\sum_{i=1}^{n-1} \left( y_{i}-1 \right)^{2}\left[ 1+10{sin}^{2}\left( {\pi y}_{i+1} \right) \right]+\left( y_{n}-1 \right)^{2} \right\}+\sum_{i=1}^{n} u\left( x_{i},10,100,4 \right)$  $y_{i}=1+\frac{x_{i}+1}{4}$  $u(x_{i},a,k,m)=\begin{matrix} \{ & \begin{matrix} k\left( x_{i}-a \right)^{m}x_{i}>a \\ 0-a<x_{i}<a \\ k\left( -x_{i}-a \right)^{m}<x_{i}<a \end{matrix} \end{matrix}$ |
| **F13** | $f\left( x \right)=0.1\left\{ {sin}^{2}\left( 3\pi x_{1} \right)+\sum_{i=1}^{n} ({x_{i}-1)}^{2}[1+{sin}^{2}\left( 3\pi x_{i}+1 \right)]+\left( x_{n}-1 \right)^{2}[1+{sin}^{2}\left( 2\pi x_{n} \right)] \right\}+\sum_{i=1}^{n} u(x_{i},5,100,4)$ |
| **F14** | $f\left( CF1 \right):$  $f_{1},f_{2},f_{3},\ldots,f_{10}=Sphere Function$  $\left[ \sigma_{1},\sigma_{2},\sigma_{3},\ldots,\sigma_{10} \right]=\left[ 1,1,1,\ldots,1 \right]$  $\left[ \lambda_{1},\lambda_{2},\lambda_{3},\ldots,\lambda_{10} \right]=\left[ \frac{5}{100},\frac{5}{100},\frac{5}{100},\ldots,\frac{5}{100} \right]$ |
| **F15** | $f\left( CF2 \right):$  $f_{1},f_{2},f_{3},\ldots,f_{10}=Griewank's Function$  $\left[ \sigma_{1},\sigma_{2},\sigma_{3},\ldots,\sigma_{10} \right]=\left[ 1,1,1,\ldots,1 \right]$  $\left[ \lambda_{1},\lambda_{2},\lambda_{3},\ldots,\lambda_{10} \right]=\left[ \frac{5}{100},\frac{5}{100},\frac{5}{100},\ldots,\frac{5}{100} \right]$ |
| **F16** | $f\left( CF3 \right):$  $f_{1},f_{2},f_{3},\ldots,f_{10}=Griewank's Function$  $\left[ \sigma_{1},\sigma_{2},\sigma_{3},\ldots,\sigma_{10} \right]=\left[ 1,1,1,\ldots,1 \right]$  $\left[ \lambda_{1},\lambda_{2},\lambda_{3},\ldots,\lambda_{10} \right]=\left[ 1,1,1,\ldots,1 \right]$ |
| **F17** | $f\left( CF4 \right):$  $f_{1},f_{2}=Ackley's Function$  $f_{3},f_{4}=Rastrigin's Function$  $f_{5},f_{6}=Weirstrass Function$  $f_{7},f_{8}=Griewank's Function$  $f_{9},f_{10}=Sphere Function$  $\left[ \sigma_{1},\sigma_{2},\sigma_{3},\ldots,\sigma_{10} \right]=\left[ 1,1,1,\ldots,1 \right]$  $\left[ \lambda_{1},\lambda_{2},\lambda_{3},\ldots,\lambda_{10} \right]=\left[ \frac{5}{32},\frac{5}{32},1,1,\frac{5}{0.5},\frac{5}{0.5},\frac{5}{100},\frac{5}{100},\frac{5}{100},\frac{5}{100} \right]$ |
| **F18** | $f\left( CF5 \right):$  $f_{1},f_{2}=Rastrigin's Function$  $f_{3},f_{4}=Weirstrass Function$  $f_{5},f_{6}= Griewank^{'}s Function$  $f_{7},f_{8}=Ackley's Function$  $f_{9},f_{10}=Sphere Function$  $\left[ \sigma_{1},\sigma_{2},\sigma_{3},\ldots,\sigma_{10} \right]=\left[ 1,1,1,\ldots,1 \right]$  $\left[ \lambda_{1},\lambda_{2},\lambda_{3},\ldots,\lambda_{10} \right]=\left[ \frac{1}{5},\frac{1}{5},\frac{5}{0.5},\frac{5}{0.5},\frac{5}{100},\frac{5}{100},\frac{5}{32},\frac{5}{32},\frac{5}{100},\frac{5}{100} \right]$ |
| **F19** | $f\left( CF6 \right):$  $f_{1},f_{2}=Rastrigin's Function$  $f_{3},f_{4}=Weirstrass Function$  $f_{5},f_{6}= Griewank^{'}s Function$  $f_{7},f_{8}=Ackley's Function$  $f_{9},f_{10}=Sphere Function$  $\left[ \sigma_{1},\sigma_{2},\sigma_{3},\ldots,\sigma_{10} \right]=\left[ 0.1,0.2,0.3,0.4,0.5,0.6,0.7,0.8,0.9,1 \right]$  $\left[ \lambda_{1},\lambda_{2},\lambda_{3},\ldots,\lambda_{10} \right]$  $=\left[ 0.1*\frac{1}{5},0.2*\frac{1}{5},0.3*\frac{5}{0.5},0.4*\frac{5}{0.5},0.5*\frac{5}{100},0.6*\frac{5}{100},0.7*\frac{5}{32},0.8*\frac{5}{32},0.9*\frac{5}{100},1*\frac{5}{100} \right]$ |
